# Systematic Review of Atrial Vascular Access for Dialysis Catheter

**DOI:** 10.1016/j.ekir.2020.04.006

**Published:** 2020-04-17

**Authors:** Carole Philipponnet, Julien Aniort, Bruno Pereira, Kazra Azarnouch, Mohammed Hadj-Abdelkader, Pascal Chabrot, Anne-Elisabeth Heng, Bertrand Souweine

**Affiliations:** 1 Nephrology, Dialysis and Transplantation Department, CHU Clermont Ferrand, Clermont Ferrand, France; 2 Department of Clinical Research and Innovation (DRCI), CHU Clermont-Ferrand, Clermont-Ferrand, France; 3 Heart Surgery Department, CHU Clermont Ferrand, Clermont Ferrand, France; 4 Department of Vascular Radiology, CHU Clermont Ferrand, Clermont Ferrand, France; 5 Medical Intensive Care Unit, UMR CNRS 6023, CHU Clermont-Ferrand, Clermont-Ferrand, France

**Keywords:** dialysis catheter, exhausted vascular accesses, hemodialysis, intra-atrial catheter

## Abstract

**Introduction:**

The last decade has seen a steady increase worldwide in the prevalence of end-stage renal disease (ESRD). Hemodialysis is the major modality of renal replacement therapy (RRT) in 70% to 90% of patients, who require well-functioning vascular access for this procedure. The recommended access for hemodialysis is an arteriovenous fistula or a vascular graft. However, recourse to central venous catheters remains essential for patients whose chronic renal disease is diagnosed at the end stage or in whom an arteriovenous fistula cannot be created or maintained. Tunneled dialysis catheter (TDC) exposure can induce venous stenosis and occlusions and can result in superior vena cava syndrome and/or vascular access loss. Exhaustion of conventional vascular accesses is 1 of the greatest challenges that nephrologists and patients have to face. Several unconventional salvage-therapy routes for TDC placement in patients with exhausted upper body venous access have been reported in the literature.

**Methods:**

We report 2 new cases of intra-atrial TDC placement for patients with exhausted vascular access and perform a meta-analysis of cases from the literature.

**Results:**

A total of 51 patients were included. The TDC was inserted by a cardiovascular surgeon in all cases. At the end of follow-up, 75% patients were alive. The median survival time was 25 months. Survival time of hemodialysis patients with intra-atrial TDC was lower than that observed with conventional TDC.

**Conclusions:**

This unconventional technique is safe and functional for hemodialysis patients with exhausted venous access. Atrial vascular access for TDC placement is salvage therapy and is therefore potentially lifesaving.

Over the past decade, there has been a steady increase in the prevalence of ESRD worldwide, with more than 2 million patients RRT. Hemodialysis is the major modality of RRT in 70% to 90% of patients, who need well-functioning vascular access for the procedure.

We report 2 hemodialysis patients with exhausted venous access who underwent atrial vascular access for TDC placement to provide RRT, in 1 case for ESRD and in the other case for prolonged acute kidney injury. We combined these 2 patient reports with all previously published cases involving a similar intravascular device to carry out a systematic meta-analysis.

## Methods

### Study Selection

The data of the 2 patients in our center with an intra-atrial hemodialysis catheter inserted between January 2010 and October 2019 were recorded. To identify relevant articles and abstracts of previously published cases of patients who had undergone the same procedure, a systematic literature search was performed using medical subject headings (MeSH) in EMBASE, CENTRAL, and MEDLINE (1980 to April 2019). The search was restricted to English-language publications involving humans. The keywords used were “renal dialysis”[MH] OR “hemodialysis”[TW] OR “kidney dialysis”[TW] OR “haemodialysis”[TW] OR “extracorporeal dialysis”[TW] OR “extracorporeal dialyses”[TW] OR “renal dialysis”[TW] OR “renal dialyses”[TW] OR “dialysis renal”[TW] OR “hemodialysis”[TW] AND “cardiac catheterization”[MH] OR “heart catheterization”[TW] OR “cardiac catheterization”[TW] OR “heart catheterizations”[TW] OR “catheterization cardiac”[TW] OR “cardiac catheterizations”[TW] OR “cardiac catheters”[MH] OR “cardiac catheter”[TW] OR “heart catheters”[TW] OR “intracardiac catheters”[TW] OR “intracardiac catheter”[TW] OR “heart catheter”[TW] OR “cardiac catheters”[TW] OR intra-atrial catheter∗[TW] OR intra-atrial[TW] AND catheter∗[TW]. We also hand-searched abstracts from international meetings.

### Inclusion and Exclusion Criteria

**Figure 1:**
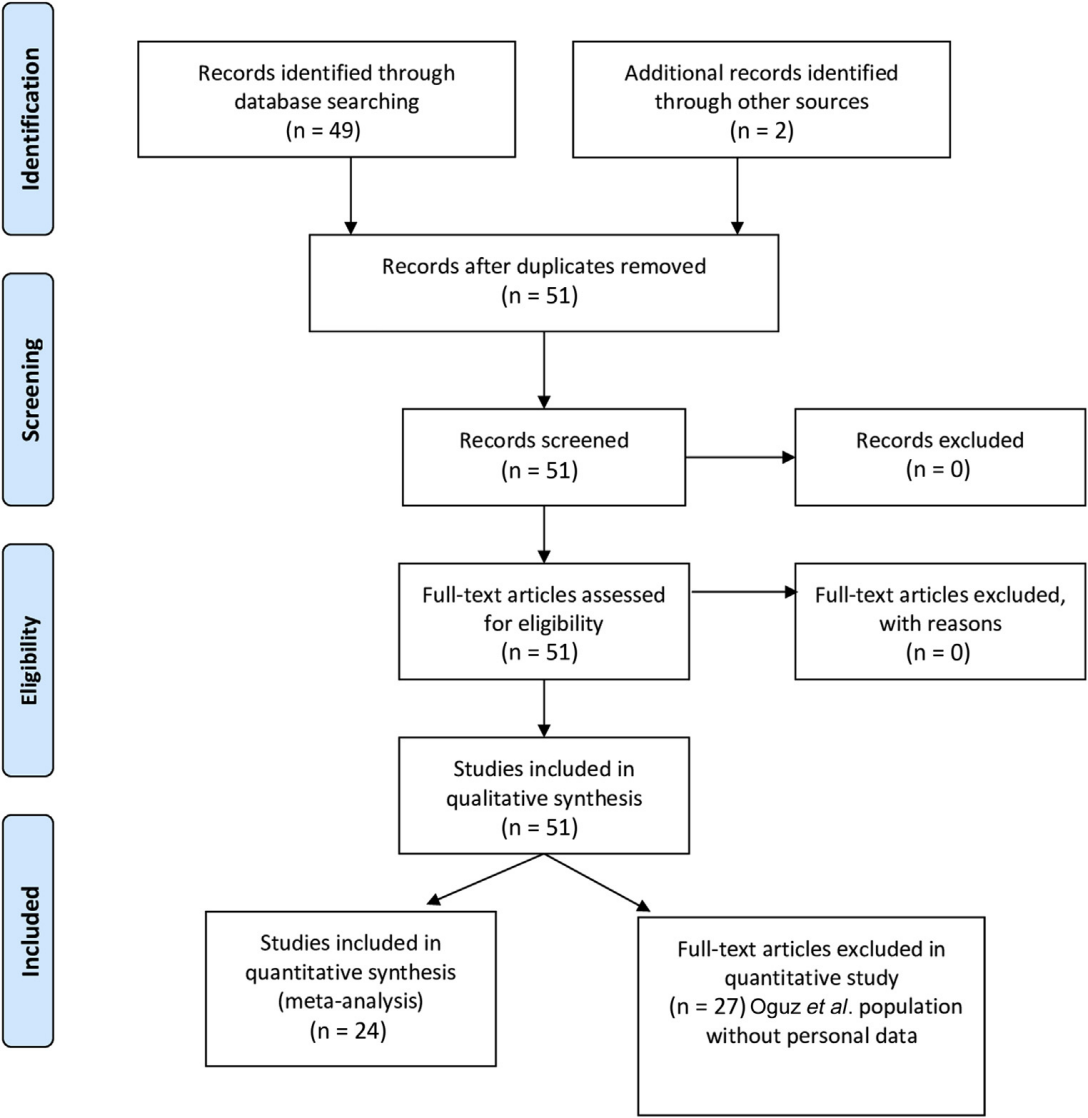
Preferred Reporting Items for Systematic Reviews and Meta-Analyses (PRISMA) flow diagram.^1^

The inclusion criteria for studies (including case reports) and patients were ESRD requiring hemodialysis and use of an intra-atrial hemodialysis catheter. The search process, eligibility assessment, and data extraction were performed independently by 2 physicians (CP and BS). The study was performed in accordance with the Preferred Reporting Items for Systematic Reviews and Meta-Analyses (PRISMA) statement. A study flow chart is provided in Figure 1^1^ and a PRISMA checklist in Supplementary File S1. The Prospero register number is CRD42019115344.

### Quality Assessment

Quality assessment was made independently by 3 investigators (CP, BS, and BP) using the framework developed by Murad *et al.* to evaluate the methodological quality of case reports/series.^2^ The level of evidence on this pyramid ranges from 0 to 10, with 5 items being measured and awarded 0, 1, or 2 points each. We assessed case and case series quality with a dedicated tool based on the criteria of Pierson, Bradford Hills, and the Newcastle−Ottawa scale.^2^ Of the 8 items, 5 were analyzed in all the case and case series, but the fourth, fifth, and sixth items were considered as not relevant (applicable to cases of adverse drug events). The items were rated on a scale of 10 points (0, not satisfactory; 1, partially satisfactory; and 2, satisfactory). The first item was considered satisfactory if the study described explicitly all the patients who had an intra-atrial TDC (IATDC) over a certain period of time. The second item was considered satisfactory if exposure was adequately ascertained. The third was considered satisfactory if outcome was adequately ascertained with catheter patency and patient outcome. The seventh item, which assesses whether follow-up was long enough for outcomes to occur, was considered satisfactory either when a competitive event occurred (kidney transplantation, switch from hemodialysis to peritoneal dialysis, or hemodialysis weaning) or when follow-up duration was at least 25 months, which corresponds to the median survival time of our study population. The seventh item was considered partially satisfactory when follow-up duration was between 7 and 24 months, with 7 months corresponding to the first interquartile of the median survival time of our study population. The last item was considered satisfactory if a surgeon could replicate the insertion procedure using the surgical description. Study quality is detailed in Supplementary File S2.

### Data Management and Statistical Analysis

Statistical analysis was performed with Stata software (version 13; StataCorp, College Station, TX). For descriptive analyses, data were presented as individual data for case reports and as median and interquartile range for case series. All analyses took into account between-study and within-study variability. To address the non-independence of data due to clustering by study, random-effects models were preferred over the usual statistical tests. The percentage of alive patients was estimated with the random-effects model as described by Der Simonian and Laird.^3^ The statistical heterogeneity in results was assessed on confidence intervals and I^2^, which quantifies inconsistency across studies describing the percentage of the variability in effect estimates that is due to heterogeneity rather than sampling error. Values of I^2^ range between 0% and 100% and are typically considered low at < 25%, moderate at 25% to 50%, and high at >50%. Overall survival was then estimated, excluding study for which individual follow-up data were not available,^1^ by the Fine and Gray method,^4^ with censoring at the date of death and at the date of kidney transplantation, switch to peritoneal dialysis, and renal recovery, defined as competing events. This analysis concerned 24 of 51 patients.

## Results

### Patients

The first patient was a 58-year-old man with mesangial IgA nephropathy requiring hemodialysis and placement of a left radiocephalic arteriovenous fistula (AVF). His medical history included ischemic cardiopathy and severe arteritis treated by aortobifemoral bypass. He underwent unsuccessful kidney transplantation, which resulted in hyperimmunization. Peritoneal dialysis was ruled out because of anuria and poor compliance.

**Figure 2:**
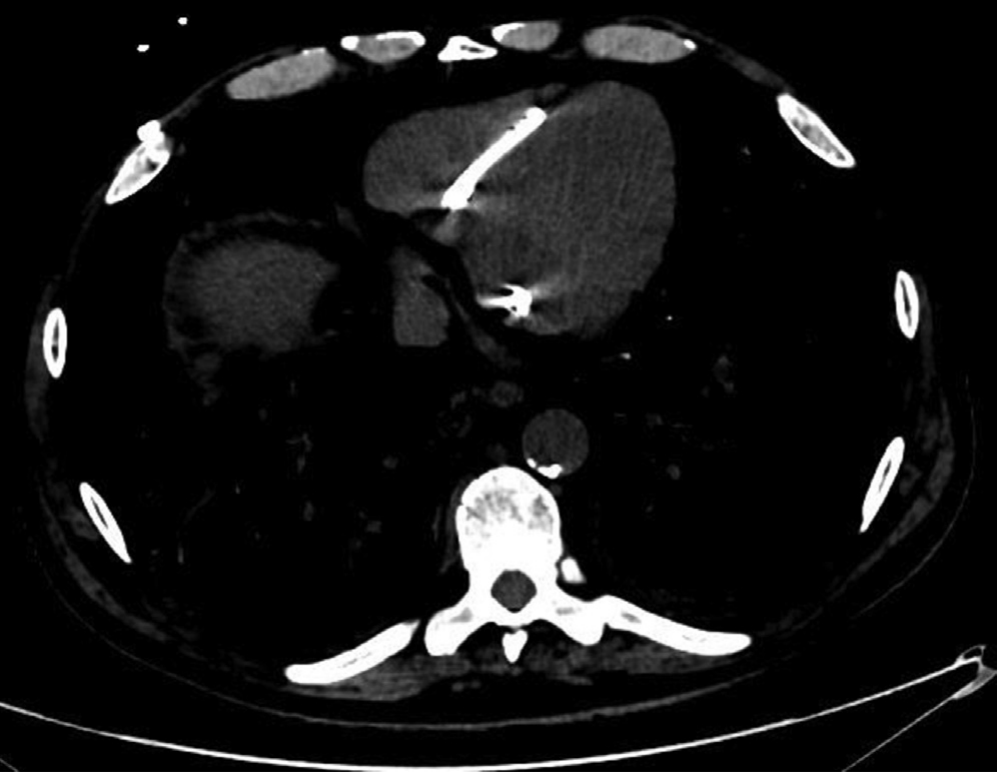
Intra-atrial tunneled dialysis catheter in patient 1.

A tunneled dialysis catheter (TDC) was placed in the right internal jugular vein after AVF thrombosis had occurred. It was removed when a left humeral−cephalic AVF was functional. The AVF required multiple angioplasties for stenosis and was ultimately occluded by thrombosis. A second TDC was placed in the left internal jugular vein. The clinical course was complicated by 5 TDC-related septic shocks, which required admission to the intensive care unit, systemic antibiotics, and a TDC replacement in the interventional radiology department after venous dilatation. All computed tomography scans and angiographies carried out to assess the vascular network showed extensive thrombosis of the brachiocephalic vein confluence and the proximal portion of the superior vena cava. The patient underwent aortic valve replacement for severe aortic stenosis and coronary artery bypass graft. During the cardiac surgery, the TDC was ablated and replaced by a new TDC directly inserted into the superior vena cava. Because of malfunction, the TDC was exchanged over a guidewire by the interventional radiologist. A new TDC-related septic shock occurred, the TDC was removed in the operating room, and systemic antibiotics were introduced for long-term treatment. A temporary hemodialysis catheter was placed at the right femoral site for 15 days and replaced by a TDC inserted directly into the right atrium by the cardiac surgeon as salvage therapy (Figure 2). Trans-lumbar, trans-hepatic, or trans-renal TDC are also salvaging approaches for vascular access. In our center, we had no experience with these techniques, and a multidisciplinary meeting retained the indication of an IATDC.

The surgical procedure was as follows: approach by right anterior thoracotomy at the third intercostal space; partial adhesiolysis to free up the right lateral pericardium surface; opening of this segment of the pericardium, and placement of 2 cuffs up against the Teflon tips on the right atrium, ready to hold the hemodialysis catheters in place; placement of Surgicel fibrillar hemostat (Johnson & Johnson Medical N.V., Diegem, Belgium) in the lumens to facilitate remote hemostasis if catheter ablation is needed; catheter tunneling via the intercostal space, just below the thoracotomy space; second tunneling path toward the lateral face of the right pectoralis major and catheter exchange over guidewire; layer-by-layer closure of the various incisions; end-of-procedure transesophageal echocardiogram to check the position of the 2 catheter tips. Any adverse events associated with IATDC catheter placement such as arrhythmia, troponin elevation, or myocardial dysfunction were noted. Nineteen months later, the patient died of metastatic bronchoalveolar carcinoma with a functional IATDC.

The second patient was a 30-year-old man with no past medical history who was admitted to the intensive care unit after a car accident that had caused multiple bone fractures, dissection of the left renal artery, and ischemic necrosis of the colon requiring multiple orthopedic surgeries and colectomy with ileostomy. Surgical and radiological attempts to restore left kidney perfusion failed. During his intensive care unit stay, the patient developed multiple episodes of septic shock that required courses of antibiotics including nephrotoxic antibiotics. He received several injections of iodinated contrast agents for diagnostic imaging and interventional radiology treatments.

Renal replacement therapy for acute kidney injury was initiated using a left internal jugular temporary hemodialysis catheter that was subsequently changed at the right and left femoral sites. All the catheters were complicated by septic thrombophlebitis and finally removed. A computed tomography scan was performed to identify an insertion site appropriate for venous access. It showed multiple thrombosis of the left internal jugular extended to the left innominate venous trunk, at the superior vena cava, the right brachiocephalic artery, and at the initial segment of the right internal jugular vein, and of the right and left iliac veins. Peritoneal dialysis was ruled out because of prior abdominal surgery. Thus, the cardiovascular surgeon placed a new TDC directly into the right atrium to allow renal RRT using the surgical procedure described above. Four months later, the patient recovered kidney function, and the IATDC was removed at the bedside without additional precautions compared to the removal of tunneled catheters. Systematic monitoring was carried after removal by ultrasound to ensure the absence of pericardial effusion. No TDC complication occurred with this last TDC. Two years later, the patient was still alive and free of dialysis.

### Literature Review

**Figure 3:**
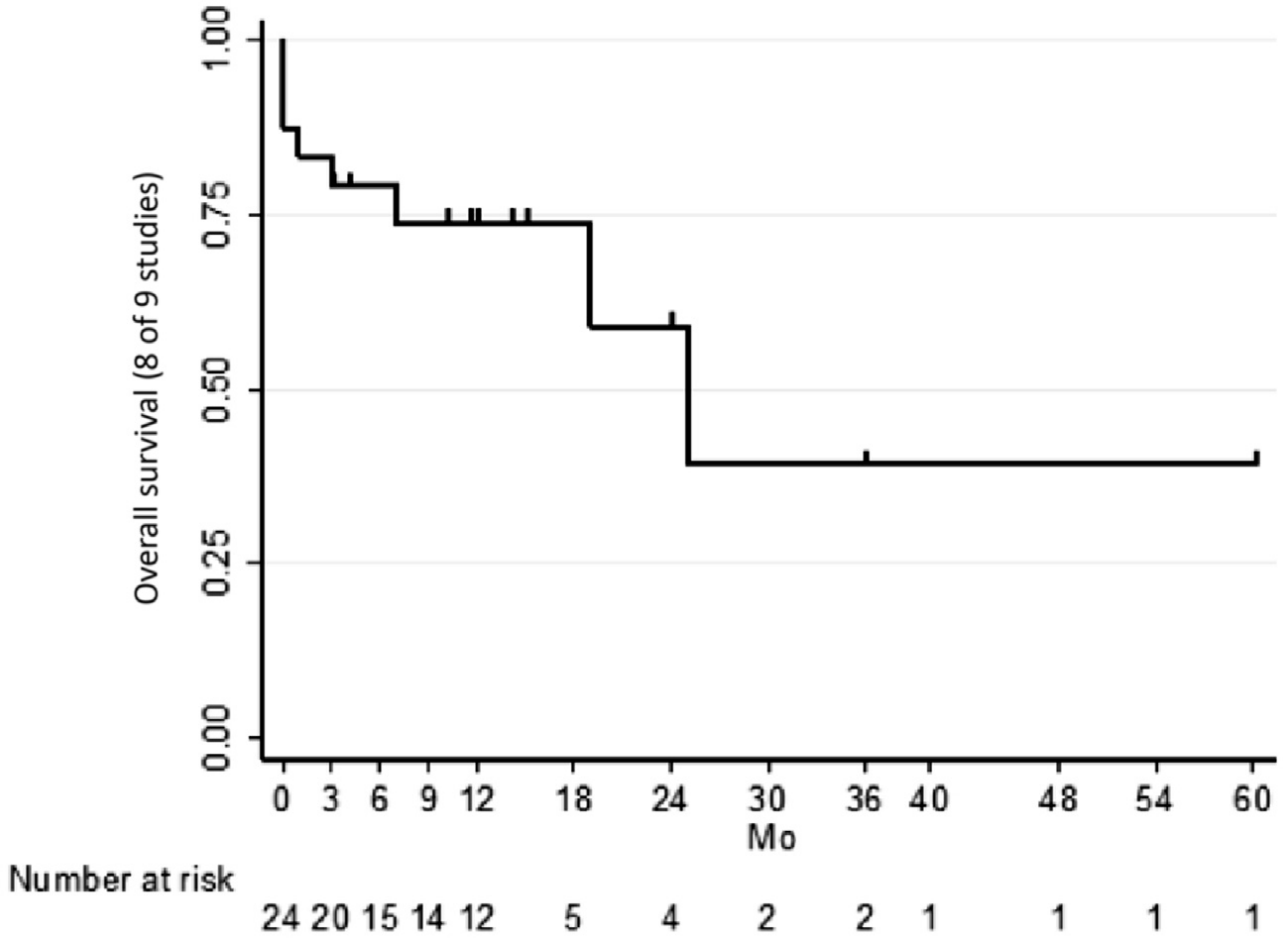
Survival time with intra-atrial tunneled dialysis catheter in 24 patients.

The above procedure of intra-atrial hemodialysis catheter insertion has been described in 4 case reports^5^, ^6^, ^7^, ^8^ and 4 small case series.^1^^,^^9^, ^10^, ^11^ The quality assessment of the studies is given in Supplementary File S2. With the addition of our 2 patients, a total of 51 patients were included in the meta-analysis. Their characteristics and outcomes are shown in Table 1.^1^^,^^5^, ^6^, ^7^, ^8^, ^9^, ^10^, ^11^ All the patients had exhausted conventional vascular accesses: they were not suitable for peritoneal dialysis or emergency kidney transplantation. All the IATDCs were inserted by a cardiovascular surgeon using the same procedure as that described in our first case report. Six patients developed IATDC-related sepsis, 1 of whom died as a result. At the end of follow-up, 38 of 51 patients were still alive (Figure 2). Seven patients died within 15 days following IATDC insertion: 3 catheter-related deaths and 4 non−catheter-related deaths (myocardial infarction, n = 2; sepsis, n = 1; metabolic, n = 1). Six additional patients died later than 15 days after IATDC insertion, from non−catheter-related sepsis (n = 2), cerebrovascular events (n = 2), neoplasia (n = 1), and unknown causes (n = 1). The 24 of 51 patients for whom individual follow-up data were available had a median survival time of 25 (7-not determined) months (Figure 3), whereas the median survival described in the Oguz *et al.* study,^1^ with no individual follow-up data available, was 27.5 ± 14.

**Table 1:** Characteristics of patients and outcomes in the different studies on IATDC

First author, year	No. of patients	Sex	Age (yr)^a^	Dialysis time (mo)^b^	Follow-up (mo)	IATDC infection/dysfunction	Outcome
Chavanon *et al.*,^5^ 1999	1	M	43	36	4	1/1	Transplantation
Santos-Araújo *et al.*,^6^ 2006	1	F	33	156	36	0/0	Pursued hemodialysis
Wales *et al.*,^7^ 2008	1	M	46	120	3	0/0	Pursued hemodialysis
Agrawal *et al.*,^10^ 2009	3	F	65	84	7	1/0	Death
Agrawal *et al.*,^10^ 2009		M	41	372	25	1/1	Death
Agrawal *et al.*,^10^ 2009		F	42	120	15	0/1	Transplantation
Villagran *et al*.,^8^ 2011	1	F	55	60	10	0/0	Pursued hemodialysis
Pereira *et al.*,^11^ 2017	7	F	76	28	0.1	1/0	Death
Pereira *et al.*,^11^ 2017		M	54	17	1.2	1/1	Death
Pereira *et al.*,^11^ 2017		F	65	149	3.3	0/1	Death
Pereira *et al.*,^11^ 2017		M	74	111	23.9	0/0	Peritoneal dialysis
Pereira *et al.*,^11^ 2017		F	69	50	0.36	0/0	Death
Pereira *et al.*,^11^ 2017		F	81	96	50	0/1	Pursued hemodialysis
Pereira *et al.*,^11^ 2017		F	44	80	11.7	1/1	Pursued hemodialysis
Yasa *et al.*,^9^ 2007	8	N/A	54 (38−66)^c^	N/A	10.2 (3−15)^c^	N/A	1 Death/7 pursued hemodialysis
Oguz *et al.*,^1^ 2012	27	10 M/17 F	59 (47−71)^c^	78.9 (33−130)^c^	N/A	0/3	5 Deaths/22 pursued hemodialysis
Philipponnet *et al.*, 2020 (current study)	2	M	30	1	4	0/0	Hemodialysis weaning
Philipponnet *et al*., 2020 (current study)		M	58	196	19	0/0	Death

IATDC, intra-atrial tunneled dialysis catheter; N/A, not available.

a Age at the time of IATDC placement.

b Time between end-stage renal disease and IATDC placement.

c Mean and SD.

## Discussion

Therapy for ESRD requires kidney transplantation or RRT, including peritoneal dialysis and hemodialysis. Hemodialysis is sometimes the only technique that can be performed in patients with contraindications to both kidney transplantation (e.g., patients with active tumor growth or very severe polyvascular disease) and peritoneal dialysis (e.g., patients with prior abdominal surgeries). In these patients, the lack of vascular access results in fatal outcomes. The recommended access for hemodialysis in ESRD patients is an AVF or a vascular graft. However, recourse to central venous catheters remains essential for patients whose chronic renal disease is diagnosed only at the end stage or in whom an arteriovenous fistula cannot be created or maintained. When it is necessary to use permanent dialysis catheters, it is recommended to use TDCs inserted in the internal jugular vein.

Exhaustion of conventional vascular accesses is 1 of the greatest challenges that nephrologist and patients have to face. Exposure of TDCs can result in venous stenosis and occlusions and superior vena cava syndrome and/or vascular access loss.^12^ Most recent works on innovations in chronic hemodialysis catheters have focused on new materials (such as carbothane and polyurethane) and new designs to prevent catheter-associated complications.^13^

Several unconventional salvage-therapy routes for TDC placement have been used in patients with no upper body venous access,^14^ including (i) needle recanalization (through a thrombosed vessel or by creating a new tract to the central vasculature through a small venous collateral or through the subcutaneous tissues),^15^^,^^16^ (ii) a translumbar approach (direct percutaneous puncture in the infrarenal inferior vena cava),^17^^,^^18^ (iii) a transhepatic approach (direct percutaneous puncture in the inferior vena cava via the right or middle hepatic vein),^19^^,^^20^ and (iv) a transrenal approach (direct percutaneous puncture in the inferior vena cava via the renal vein).^21^^,^^22^ Intra-atrial placement is an alternative strategy.

To the best of our knowledge, the present study collates all published cases of patients with an IATDC. As shown, IATDC exchange or removal for dysfunction, thrombosis, or infection was scarce, suggesting that IATDC patency was good, that it achieved adequate blood flow rates, and that IATDC placement was associated with prolonged survival.

In the US Renal Data System, approximately 510,000 ESRD patients initiated hemodialysis between 2006 and 2010. Of the 82.5% patients receiving dialysis with a TDC, 78% had 1-year survival and 45% 5 year-survival with a median survival time of 3 years.^23^ In our meta-analysis, median survival time in patients with an IATDC was 25 (7-NA) months (Figure 3). These findings suggest that the survival time observed in patients with an IATDC is lower than in patients with conventional TDC. However, the population with an IATDC formed a subgroup of patients with highly severe comorbid conditions.

Our study has several strengths. First, we performed a quality assessment of cases and case series with a validated tool.^2^ Second, we identified for the first time all patients with an IATDC and described the successful use of an unrecognized technique as salvage therapy.

Our study also has several major limitations. First, the patients were retrospectively identified in our center and in documented reports, and hence we cannot rule out the possibility that some with IATDC were not included. However, a randomized controlled trial would have been impossible because there was no other alternative therapy. Second, only 51 patients were included in the analysis. At the same time, however, IATDC is a very rare procedure. Third, individual data from 1 case series were not available, and hence these patients could not be included in the Kaplan−Meier analysis.^1^

In conclusion, IATDC is an unconventional but safe procedure for adequate vascular access in hemodialysis patients with exhausted venous access. The technique requires a collaborative multidisciplinary approach involving a radiologist, cardiac surgeon, and nephrologist. Atrial vascular access for TDC placement can potentially be lifesaving.

## Disclosure

All the authors declared no competing interests.
