# Supplementary material for: Systematic Review of Atrial Vascular Access for Dialysis Catheter
Source: Kidney Int Rep. 2020 Apr 17;5(7):1000–6. doi: 10.1016/j.ekir.2020.04.006 (PMC7335951; doi:10.1016/j.ekir.2020.04.006)
Supplement: Supplementary File (PDF) [file mmc1.pdf]

Section/topic	#	Checklist item	Reported on page #
**TITLE**			
Title	1	Identify the report as a systematic review, meta-analysis, or both.	1
**ABSTRACT**			
Structured summary	2	Provide a structured summary including, as applicable: background; objectives; data sources; study eligibility criteria, participants, and interventions; study appraisal and synthesis methods; results; limitations; conclusions and implications of key findings; systematic review registration number.	2
**INTRODUCTION**			
Rationale	3	Describe the rationale for the review in the context of what is already known.	3
Objectives	4	Provide an explicit statement of questions being addressed with reference to participants, interventions, comparisons, outcomes, and study design (PICOS).	3
**METHODS**			
Protocol and registration	5	Indicate if a review protocol exists, if and where it can be accessed (e.g., Web address), and, if available, provide registration information including registration number.	3-4
Eligibility criteria	6	Specify study characteristics (e.g., PICOS, length of follow-up) and report characteristics (e.g., years considered, language, publication status) used as criteria for eligibility, giving rationale.	3-4
Information sources	7	Describe all information sources (e.g., databases with dates of coverage, contact with study authors to identify additional studies) in the search and date last searched.	3-4
Search	8	Present full electronic search strategy for at least one database, including any limits used, such that it could be repeated.	3-4
Study selection	9	State the process for selecting studies (i.e., screening, eligibility, included in systematic review, and, if applicable, included in the meta-analysis).	3-4
Data collection process	10	Describe method of data extraction from reports (e.g., piloted forms, independently, in duplicate) and any processes for obtaining and confirming data from investigators.	3-4
Data items	11	List and define all variables for which data were sought (e.g., PICOS, funding sources) and any assumptions and simplifications made.	3-4
Risk of bias in individual studies	12	Describe methods used for assessing risk of bias of individual studies (including specification of whether this was done at the study or outcome level), and how this information is to be used in any data synthesis.	3-4
Summary measures	13	State the principal summary measures (e.g., risk ratio, difference in means).	3-4
Synthesis of results	14	Describe the methods of handling data and combining results of studies, if done, including measures of consistency (e.g., I^2) for each meta-analysis.	3-4

Section/topic	#	Checklist item	Reported on page #
Risk of bias across studies	15	Specify any assessment of risk of bias that may affect the cumulative evidence (e.g., publication bias, selective reporting within studies).	3-4
Additional analyses	16	Describe methods of additional analyses (e.g., sensitivity or subgroup analyses, meta-regression), if done, indicating which were pre-specified.	3-4
**RESULTS**			
Study selection	17	Give numbers of studies screened, assessed for eligibility, and included in the review, with reasons for exclusions at each stage, ideally with a flow diagram.	5-7
Study characteristics	18	For each study, present characteristics for which data were extracted (e.g., study size, PICOS, follow-up period) and provide the citations.	5-7
Risk of bias within studies	19	Present data on risk of bias of each study and, if available, any outcome level assessment (see item 12).	5-7
Results of individual studies	20	For all outcomes considered (benefits or harms), present, for each study: (a) simple summary data for each intervention group (b) effect estimates and confidence intervals, ideally with a forest plot.	5-7
Synthesis of results	21	Present results of each meta-analysis done, including confidence intervals and measures of consistency.	5-7
Risk of bias across studies	22	Present results of any assessment of risk of bias across studies (see Item 15).	5-7
Additional analysis	23	Give results of additional analyses, if done (e.g., sensitivity or subgroup analyses, meta-regression [see Item 16]).	5-7
**DISCUSSION**			
Summary of evidence	24	Summarize the main findings including the strength of evidence for each main outcome; consider their relevance to key groups (e.g., healthcare providers, users, and policy makers).	7-9
Limitations	25	Discuss limitations at study and outcome level (e.g., risk of bias), and at review-level (e.g., incomplete retrieval of identified research, reporting bias).	9
Conclusions	26	Provide a general interpretation of the results in the context of other evidence, and implications for future research.	9
**FUNDING**			
Funding	27	Describe sources of funding for the systematic review and other support (e.g., supply of data); role of funders for the systematic review.	10

Supplementary file 1 S1 : Prisma checklist

Supplemental file 2 S2: Quality assessment of the studies

	Item1	Item2	Item3	Item4	Item5	Item6	Item7	Item8	Total Score /10
Yasa 2007	2	2	2	NA	NA	NA	1	2	9
Oguz 2012	2	2	2	NA	NA	NA	2	2	10
Pereira 2017	2	2	2	NA	NA	NA	2	2	10
Chavanon 1999	0	2	2	NA	NA	NA	2	1	7
Agrawal 2009	2	2	2	NA	NA	NA	2	2	10
Villagran 2011	0	2	2	NA	NA	NA	1	2	7
Santos Araujo 2006	0	2	2	NA	NA	NA	2	1	7
Wales 2008	0	2	2	NA	NA	NA	0	2	6
Philipponnet 2020	2	2	2	NA	NA	NA	2	2	10

Item 1, selection1; Does the patient(s) represent(s) the whole experience of the investigator (centre) or is the selection method unclear to the extent that other patients with similar presentation may not have been reported?
Item 2, ascertainment2; Ascertainment 2. Was the exposure adequately ascertained?
Item 3, ascertainment3; Ascertainment 3. Was the outcome adequately ascertained?
Item 4, causality 4; Were other alternative causes that may explain the observation ruled out?
Item 5 causality 5; Was there a challenge/rechallenge phenomenon?
Item 6, causality 6; Was there a dose–response effect?
Item 7, causality7; Was follow-up long enough for outcomes to occur?
tem 8, reporting 8; Is the case(s) described with sufficient details to allow other investigators to replicate the research or to allow practitioners make inferences related to their own practice?

0 : not satisfactory
1 : partially satisfactory
2 : satisfactory
NA : Not applicable

Yasa 2007

Selection 1. Does the patient(s) represent(s) the whole experience of the investigator (centre) or is the selection method unclear to the extent that other patients with similar presentation may not have been reported?
All 8 hemodialysis patients fitted with an intra-atrial tunneled dialysis catheter (IATDC) between March 2003 and August 2005 in the cardiovascular surgery department in Izmir are reported.

Ascertainment 2. Was the exposure adequately ascertained?

The histories of the 8 patients are given with details of exhausted vascular access.

Ascertainment 3. Was the outcome adequately ascertained?
The outcomes of the 8 patients are given: 1 death and 7 hemodialysis with IATDC.

Causality 4. Were other alternative causes that may explain the observation ruled out?
Not applicable.

Causality 5. Was there a challenge/rechallenge phenomenon?
Not applicable.

Causality 6. Was there a dose–response effect?
Not applicable.

Causality 7. Was follow-up long enough for outcomes to occur?
The mean follow-up was 10 months (range 3 to 15).

Reporting 8. Is the case(s) described with sufficient details to allow other investigators to replicate the research or to allow practitioners make inferences related to their own practice?
The surgical technique is described in full.

Oguz 2012

Selection 1. Does the patient(s) represent(s) the whole experience of the investigator (centre) or is the selection method unclear to the extent that other patients with similar presentation may not have been reported?
All 27 hemodialysis patients fitted with an intra-atrial tunneled dialysis catheter (IATDC) between October 2005 and October 2010 in the cardiovascular surgery department in Izmir are reported.

Ascertainment 2. Was the exposure adequately ascertained?
The histories of the 27 patients are given with details of exhausted vascular access

Ascertainment 3. Was the outcome adequately ascertained?
The outcomes of the 27 patients are described: 5 deaths and 22 hemodialysis with IATDC

Causality 4. Were other alternative causes that may explain the observation ruled out?
Not applicable

Causality 5. Was there a challenge/rechallenge phenomenon?
Not applicable

Causality 6. Was there a dose–response effect?
Not applicable

Causality 7. Was follow-up long enough for outcomes to occur?
The follow-up was more than 27 months

Reporting 8. Is the case(s) described with sufficient details to allow other investigators to replicate the research or to allow practitioners make inferences related to their own practice?
The surgical technique is well described.

Pereira 2016

Selection 1. Does the patient(s) represent(s) the whole experience of the investigator (centre) or is the selection method unclear to the extent that other patients with similar presentation may not have been reported?
All 7 hemodialysis patients fitted with an intra-atrial tunneled dialysis catheter (IATDC) between January 2004 and December 2015 in the cardiovascular surgery department in the Santa Maria hospital, Lisbon, are reported.

Ascertainment 2. Was the exposure adequately ascertained?
The histories of the 7 patients are given with details of exhausted vascular access

Ascertainment 3. Was the outcome adequately ascertained?
The outcomes of the 7 patients are given: 4 deaths, 1 peritoneal dialysis and 2 hemodialysis with IATDC

Causality 4. Were other alternative causes that may explain the observation ruled out?
Not applicable

Causality 5. Was there a challenge/rechallenge phenomenon?
Not applicable

Causality 6. Was there a dose–response effect?
Not applicable

Causality 7. Was follow-up long enough for outcomes to occur?
The follow-up was more than 12 years

Reporting 8. Is the case(s) described with sufficient details to allow other investigators to replicate the research or to allow practitioners make inferences related to their own practice?
The surgical technique is well described.

Chavanon 1999

Selection 1. Does the patient(s) represent(s) the whole experience of the investigator (centre) or is the selection method unclear to the extent that other patients with similar presentation may not have been reported?
A single patient with IATDC was reported.

Ascertainment 2. Was the exposure adequately ascertained?
The history of the patient is given with details of exhausted vascular access

Ascertainment 3. Was the outcome adequately ascertained?
The outcome of the patient is given: transplantation

Causality 4. Were other alternative causes that may explain the observation ruled out?
Not applicable

Causality 5. Was there a challenge/rechallenge phenomenon?
Not applicable

Causality 6. Was there a dose–response effect?
Not applicable

Causality 7. Was follow-up long enough for outcomes to occur?
The follow-up was until transplantation

Reporting 8. Is the case(s) described with sufficient details to allow other investigators to replicate the research or to allow practitioners make inferences related to their own practice?
The surgical technique is briefly described.

Agrawal 2009

Selection 1. Does the patient(s) represent(s) the whole experience of the investigator (centre) or is the selection method unclear to the extent that other patients with similar presentation may not have been reported?
All 3 hemodialysis patients fitted with an intra-atrial tunneled dialysis catheter (IATDC) between 2003 and 2009 in the nephrology department, Columbia, are reported.

Ascertainment 2. Was the exposure adequately ascertained?
The histories of the 3 patients are given with details of exhausted vascular access

Ascertainment 3. Was the outcome adequately ascertained?
The outcomes of the 3 patients are given: 2 deaths and 1 transplantation

Causality 4. Were other alternative causes that may explain the observation ruled out?
Not applicable

Causality 5. Was there a challenge/rechallenge phenomenon?
Not applicable

Causality 6. Was there a dose–response effect?
Not applicable

Causality 7. Was follow-up long enough for outcomes to occur?
The follow-up was until death (2) and transplantation (1)

Reporting 8. Is the case(s) described with sufficient details to allow other investigators to replicate the research or to allow practitioners make inferences related to their own practice?
The surgical technique is well described.

Villagran 2010

Selection 1. Does the patient(s) represent(s) the whole experience of the investigator (centre) or is the selection method unclear to the extent that other patients with similar presentation may not have been reported?
A single patient with IATDC was reported.

Ascertainment 2. Was the exposure adequately ascertained?
The history of the patient is given with details of exhausted vascular access

Ascertainment 3. Was the outcome adequately ascertained?
The outcome of the patient is given: hemodialysis with IATDC

Causality 4. Were other alternative causes that may explain the observation ruled out?
Not applicable

Causality 5. Was there a challenge/rechallenge phenomenon?
Not applicable

Causality 6. Was there a dose–response effect?
Not applicable

Causality 7. Was follow-up long enough for outcomes to occur?
The follow-up was 10 months

Reporting 8. Is the case(s) described with sufficient details to allow other investigators to replicate the research or to allow practitioners make inferences related to their own practice?
The surgical technique is well described.

Santos Araujo 2006

Selection 1. Does the patient(s) represent(s) the whole experience of the investigator (centre) or is the selection method unclear to the extent that other patients with similar presentation may not have been reported?
A single patient with intra atrial was reported.

Ascertainment 2. Was the exposure adequately ascertained?
The history of the patient is given with details of exhausted vascular access

Ascertainment 3. Was the outcome adequately ascertained?
The outcome of the patient is given: hemodialysis with IATDC

Causality 4. Were other alternative causes that may explain the observation ruled out?
Not applicable

Causality 5. Was there a challenge/rechallenge phenomenon?

Not applicable

Causality 6. Was there a dose–response effect?
Not applicable

Causality 7. Was follow-up long enough for outcomes to occur?
The follow-up was 36 months

Reporting 8. Is the case(s) described with sufficient details to allow other investigators to replicate the research or to allow practitioners make inferences related to their own practice?
The surgical technique is briefly described.

Wales 2008

Selection 1. Does the patient(s) represent(s) the whole experience of the investigator (centre) or is the selection method unclear to the extent that other patients with similar presentation may not have been reported?
A single patient with IATDC was reported.

Ascertainment 2. Was the exposure adequately ascertained?
The history of the patient is given with details of exhausted vascular access

Ascertainment 3. Was the outcome adequately ascertained?
The outcome of the patient is given: hemodialysis with IATDC

Causality 4. Were other alternative causes that may explain the observation ruled out?
Not applicable

Causality 5. Was there a challenge/rechallenge phenomenon?
Not applicable

Causality 6. Was there a dose–response effect?
Not applicable

Causality 7. Was follow-up long enough for outcomes to occur?
The follow up was 3 months

Reporting 8. Is the case(s) described with sufficient details to allow other investigators to replicate the research or to allow practitioners make inferences related to their own practice?
The surgical technique is well described.
